# Medical Items

**Published:** 1914-02

**Authors:** 


### MEDICAL ITEMS

### PIONEERS.

As a result of the newspaper notoriety given bichloride of having pulmonary tuberculosis reveals the presence or garding the sale of tablets containing this substance. It is a dangerous poison and one that should be surrounded with every safeguard to prevent accidental misuse and error. That evidently was the idea in mind when Diamond Antiseptics were placed on the market nine years ago. These tablets were pioneer safeguards then and have continued to minimize the dangers to users of bichloride ever since. Diamond Antiseptics render legal enactments unnecessary. The talk of legislation and restrictions has also led other manufacturers to offer bichloride of mercury tablets of special design supplied in special containers so that if legislation of any kind is needed, all that should be done it would seem, is to prohibit the sale of bichloride of mercury in tablet form in the shape of the ordinary round or cylindrical medicinal tablet. The original pioneer safeguards, however, were and are Diamond Antiseptics, Lilly.

